# Supplementary material for: Malaria parasite CelTOS targets the inner leaflet of cell membranes for pore-dependent disruption
Source: eLife. 2016 Dec 1;5:e20621. doi: 10.7554/eLife.20621 (PMC5132341; doi:10.7554/eLife.20621)
Supplement: Figure 1—source data 1. — DOI: http://dx.doi.org/10.7554/eLife.20621.004 [file elife-20621-fig1-data1.rtf]

Supplementary information: source data, supplementary files and supplementary figures.
Figure 1—source data 1.  Data collection, phasing and refinement statistics 

	Native	Hg derivative	Pt derivative	
Data collection				
Space group	P 31 2 1	P 31 2 1	P 31 2 1	
Cell dimensions  				
    a, b, c (Å)	158.96, 158.96, 64.09	158.66, 158.66, 63.85	158.00, 158.00, 63.43	
    a, b, g  () 	90, 90, 120	90, 90, 120	90, 90, 120	
Resolution (Å)	20.0 – 3.0 (3.1 – 3.0)	20.0 – 3.9 (4.0 – 3.9)	20.0 – 4.1 (4.2 – 4.1)	
Rsym (%)	9.6 (89.9)	17.9 (90.8)	29.5 (79.0)	
I / sI	14.9 (1.7)	17.9 (6.9)	17.3 (7.59)	
Completeness (%)	96.6 (92.9)	99.8 (99.2)	99.9 (100.0)	
Redundancy	5.0 (4.9)	28.6 (28.0)	28.6 (28.5)	
				
Refinement				
Resolution (Å)	20.0 – 3.0			
No. reflections	18,208			
Rwork / Rfree	21.97 / 23.78			
No. atoms				
    Protein	3,351			
    Ligand/ion	3			
    Water	9			
B-factors				
    Protein	73.6			
    Ligand/ion	64.0			
    Water	79.4			
R.m.s. deviations				
    Bond lengths (Å)	0.007			
    Bond angles ()	0.690			
Each data set was collected from a single crystal.
Highest resolution shell is shown in parenthesis.
