# Supplementary material for: Malaria parasite CelTOS targets the inner leaflet of cell membranes for pore-dependent disruption
Source: eLife. 2016 Dec 1;5:e20621. doi: 10.7554/eLife.20621 (PMC5132341; doi:10.7554/eLife.20621)
Supplement: Figure 1—source data 2. — Three independent global fits for three concentrations and two speeds demonstrate Pf and PvCelTOS are dimers in solution. The theoretical monomer molecular weight for PfCelTOS and PvCelTOS are 18.655 kDa and 18.665 kDa, respectively. DOI: http://dx.doi.org/10.7554/eLife.20621.005 [file elife-20621-fig1-data2.rtf]

Figure 1—source data 2. Sedimentation equilibrium analytical ultracentrifugation analysis for Pf and PvCelTOS. Three independent global fits for three concentrations and two speeds demonstrate Pf and PvCelTOS are dimers in solution. The theoretical monomer molecular weight for PfCelTOS and PvCelTOS are 18.655 kDa and 18.665 kDa, respectively.

Protein	Concentrations [µM]	Speeds
(rpm)	Observed Molecular
Weight [kDa]	Variance
(×10-5)	Oligomeric State	Average Molecular Weight [kDa] ± s.d.	
PfCelTOS	46, 36, 25	12k, 15k	37.4	1.36	Dimer		
PfCelTOS	46, 36, 25	12k, 15k	38.4	1.64	Dimer	37.67 ± 0.64	
PfCelTOS	46, 36, 25	12k, 15k	37.2	1.9	Dimer		
							
PvCelTOS	53, 41, 29	12k, 15k	39.3	1.53	Dimer		
PvCelTOS	53, 41, 29	12k, 15k	37.7	1.69	Dimer	38.23 ± 0.92	
PvCelTOS	53, 41, 29	12k, 15k	37.7	1.59	Dimer		
